# Circulating exosomal PCAT1 as a complement of carcinoembryonic antigen for early colorectal cancer diagnosis

**DOI:** 10.1016/j.heliyon.2024.e39264

**Published:** 2024-10-11

**Authors:** Jinghe Cao, Wei Chao, Jiansheng Zhang, Jiajia Mao, Jianchao Zeng, Delan Luo, Shishun Huang, Jiashu Li, Baoyu He, Hongli Pan

**Affiliations:** a Department of Laboratory Medicine, Affiliated Hospital of Jining Medical University, Jining Medical University, Shandong, China; b Medical Science Laboratory, the Fourth Affiliated Hospital of Guangxi Medical University, Guangxi, China; c Medical Science Experimental Center of Guangxi Medical University, Guangxi, China; d Department of Scientific Research and Education, the First Affiliated Hospital of Guangxi Medical University, Guangxi, China; e Department of Gastroenterology, the First People's Hospital of Neijiang City, Sichuan, China; f Department of Pathology, the First Affiliated Hospital of Guangxi Medical University, Guangxi, China

**Keywords:** Serum exosomes, PCAT1, Carcinoembryonic antigen, Colorectal cancer, Complementary biomarker

## Abstract

**Backgrounds:**

Given the global prevalence of colorectal cancer (CRC), advancements in prompt and accurate diagnosis are crucial. Long non-coding RNAs (lncRNAs) in serum exosomes are emerging as potential diagnostic biomarkers. This study evaluated the feasibility of using serum exosomal lncRNAs for early-stage CRC diagnosis in clinical practice.

**Methods:**

Candidate serum exosomal lncRNAs were identified through an integrated analysis of two GEO datasets (GSE100206 and GSE100063) containing non-coding RNA expression profiles in serum exosomes. Exosomes isolated from participants' serum were validated using transmission electron microscopy (TEM) and immunoblotting. The expression levels of serum exosomal PCAT1 were measured by quantitative real-time polymerase chain reaction (qRT-PCR). Diagnostic accuracy was assessed using receiver operating characteristic (ROC) analysis.

**Results:**

Serum exosomal PCAT1 levels were evaluated in 150 CRC patients, 66 patients with benign colorectal lesions, and 128 healthy controls. ROC analysis demonstrated high diagnostic efficacy of serum exosomal PCAT1 for CRC. Notably, the predictive performance was sufficient to distinguish early-stage CRC patients. Additionally, the diagnostic value was significant for CRC patients with low serum carcinoembryonic antigen (CEA) levels. Measuring serum exosomal PCAT1 could complement CEA assessment, enhancing CRC diagnostic accuracy.

**Conclusions:**

Serum exosomal PCAT1 can complement CEA assessment, aiding in early CRC diagnosis and helping to differentiate the disease, especially in patients with low CEA levels.

## Highlights

• LncRNA PCAT1 is stably located in serum exosomes with high abundance from CRC patients.
• Serum exosomal PCAT1 level is substantially elevated in patients with early-stage CRC.
• Serum exosomal PCAT1 can act as a complementary biological marker for CEA in CRC early diagnosis.
• Serum exosomal PCAT1 level decreases in post-surgical resection of CRC patients.

## 1 Introduction

Colorectal cancer (CRC) is the third most common cancer and the second leading cause of cancer-related deaths worldwide [1,2], accounting for approximately 9 % of all cancer fatalities [[1], [2], [3]]. The 5-year survival rate for patients with localized CRC is up to 90 %, but it drops to 70 % with regional metastasis and 15 % with distant metastases [[3], [4], [5]]. Thus, early detection and screening are essential for improving long-term survival. However, traditional stool-based tests lack sensitivity and specificity, and colonoscopy, though effective, is costly and has low compliance [1,6,7]. Carcinoembryonic antigen (CEA) is the most commonly used serum biomarker for CRC, but its sensitivity ranges from 35 to 75 % at the standard threshold of 3.08 g/L, and is especially poor for early-stage CRC (20–60 %) [8,9]. Additionally, elevated CEA levels are found in many patients with non-malignant conditions such as colon polyps (12–25 %) and Crohn's disease (10–22 %) [10,11]. These limitations highlight the need for additional robust diagnostic markers to complement CEA.

Long non-coding RNAs (lncRNAs) are extensively expressed and play crucial roles in regulating physiological processes in various cells and tissues [[12], [13], [14]]. Recent studies have suggested circulating lncRNAs in serum as promising biomarkers [15]. Our previous research demonstrated that a three-lncRNA signature in serum could effectively distinguish early nasopharyngeal cancer from chronic nasopharyngitis, showing superior diagnostic performance [16]. The lncRNA LOC553103 also exhibited strong diagnostic capabilities across 15 solid cancer types [17]. Exosomes, small membrane vesicles (30–120 nm) secreted by various cells and found in body fluids, reflect the molecular composition of their originating tumor cells, containing proteins, DNA, RNA, and lipids [[18], [19], [20], [21]]. Exosomal lncRNAs have emerged as powerful, non-invasive markers for cancer diagnosis and prognosis [22,23].

In this study, we utilized bioinformatics analyses and laboratory experiments to identify serum exosomal lncRNA biomarkers that could enhance early CRC diagnosis alongside CEA. Through a stepwise screening and validation process, we identified a potential serum exosomal lncRNA biomarker for early-stage CRC detection. Additionally, we assessed the utility of PCAT1 in predicting tumor progression and treatment efficacy.

## 2 Methods

### 2.1 Study design

**Fig. 1:**
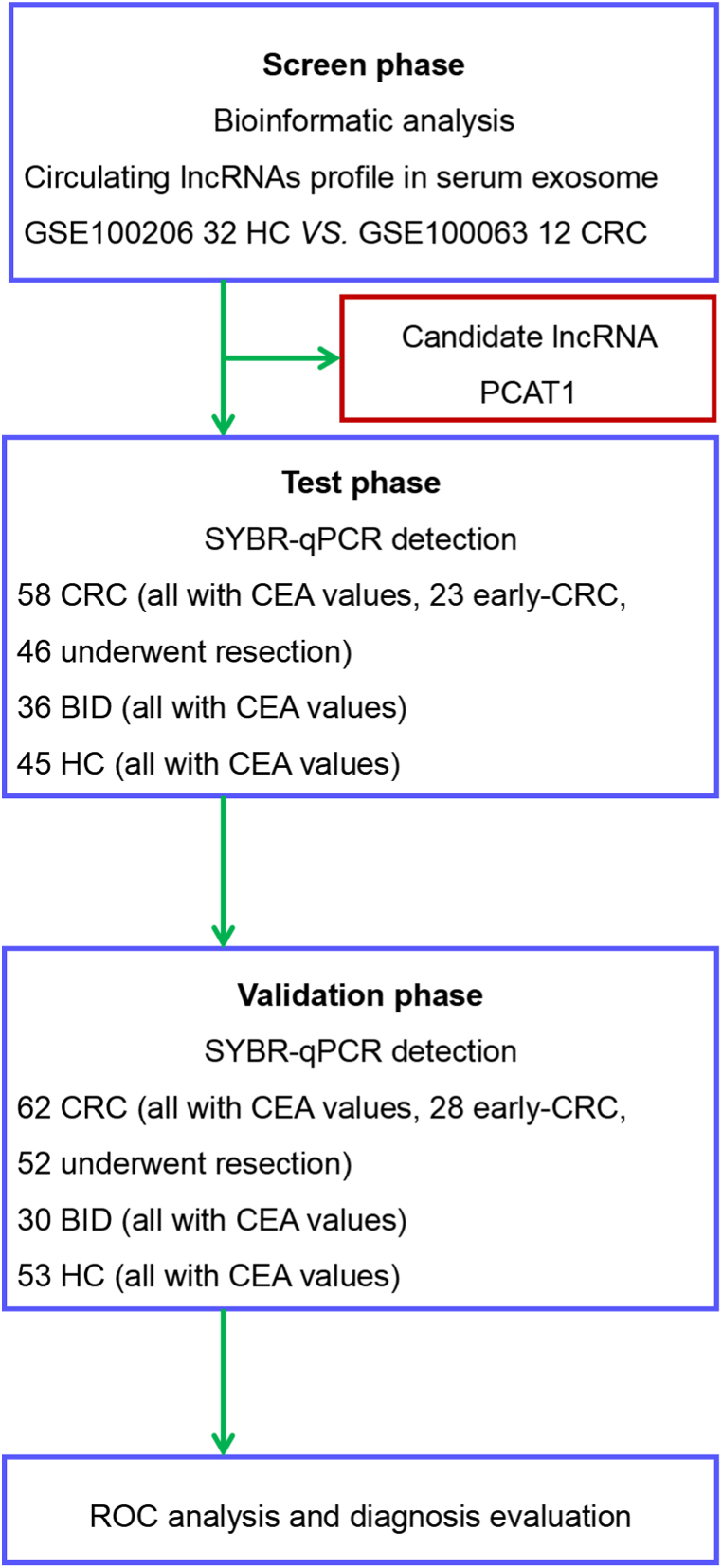
Study overview. A three-stage method was employed to systematically screen and validate serum exosomal lncRNA biomarkers for the early diagnosis of CRC.

As shown in Fig. 1, the diagnostic potential of candidate lncRNAs for early-stage CRC was systematically investigated through a three-phase approach.

In the screening phase, two independent datasets of serum exosomal non-coding RNA profiles from 32 healthy individuals (GSE100206) and 12 CRC patients (GSE100063) were analyzed. Fifty-three differentially expressed serum exosomal lncRNAs were identified as preliminary biomarkers (Supplemental Table S1).

In the testing and validation phase, serum samples from two additional independent cohorts—120 CRC patients, 66 with benign colorectal lesions (BID), and 98 healthy controls—were analyzed to further evaluate PCAT1 levels. The test cohort included 45 HC, 36 patients with BID, and 58 CRC patients. The test cohort was recruited from May 2018 to July 2020. The validation cohort comprised 53 HC, 30 BID, and 62 CRC patients. The validation cohort was recruited from August 2020 to June 2022. The diagnostic significance of serum exosomal PCAT1 was determined by comparing its relative expression across these cohorts with that of the controls.

### 2.2 Study population

Patients with CRC (n = 150), BID (n = 66), and healthy controls (HCs, n = 128) were recruited from the Fourth Affiliated Hospital of Guangxi Medical University. One hundred and fifty subjects were diagnosed as CRC through colonoscopy, abdominal ultrasonography, clinical evaluation and histopathology confirmation. Thirty-two patients with metastatic disease, those who received neoadjuvant therapy, those who received systemic chemoradiotherapy, those who had any known systemic inflammatory or autoimmune disease, those with another concurrent malignancy and those who had been previously diagnosed and treated for another malignancy were excluded from the study. Fifty-one subjects with Dukes stage A or B tumors were categorized as early-stage CRC due to the absence of lymphatic or vascular metastases, corresponding to tumor stages I or II. The BID group included 66 patients with Crohn's disease, intestinal obstruction, colorectal hyperplastic polyp, and acute or chronic enteritis. Healthy controls were selected from disease-free volunteers age and sex-matched with the patients. Recruitment took place from May 2018 to June 2022.

Peripheral blood samples were collected in anticoagulant-free tubes at diagnosis, then centrifuged and stored at −80 °C for future analysis. Venous blood was drawn before surgical resection for diagnostic assessment. After surgical resection, 212 serum samples from 120 CRC patients were collected to evaluate therapy effectiveness. Efforts were made to match CRC patients and HCs as closely as possible by sex and age. Data collection and analysis were carried out independently by three researchers. Clinical and pathological details of participants are summarized in Supplemental Table S2.

### 2.3 Transmission electron microscopy (TEM)

The methods for TEM were performed as described previously [24]. Exosomes were suspended in PBS and applied to carbon-coated copper grids. After a 10-min incubation, the grids were stained with 3 % glutaraldehyde for 5 min. The grids were then washed with distilled water. Following this, staining with 4 % uranyl acetate and 1 % methyl cellulose was carried out, and the grids were allowed to dry. The exosome morphologies were examined using a transmission electron microscope (H-7650, Japan).

### 2.4 Total exosomal RNA extraction and quantitative RT-PCR

Total exosomal RNA was extracted from serum samples using the miRNeasy Serum/Plasma Kit (Qiagen) as per the manufacturer's protocol. The pGL3 plasmid was added to the serum samples to serve as an internal control, given the absence of appropriate serological controls. Specifically, 1 ng of pGL3 (approximately 2 × 10^8^ copies) was introduced into 200 μL of serum to enhance data reliability. Reverse transcription was performed on 1 μg of RNA using the 1st-Strand cDNA Synthesis Kit (LM-63826, LMAI-BIO). Quantitative polymerase chain reaction (qPCR) was carried out with SYBR Green using iTaq Universal SYBR Green Supermix (Bio-Rad, Hercules, CA), while TaqMan-based qPCR was performed using iTaq Universal Probes Supermix (Bio-Rad, Hercules, CA). Fold changes in PCAT1 expression were determined using formula 2^−ΔΔCt^.

### 2.5 Statistical analysis

Statistical analyses were performed using SPSS 13.0 and GraphPad Prism 5.0. Comparisons of serum exosomal PCAT1 levels between CRC patients and healthy controls were conducted with Student's *t*-tests. The Chi-square test was used to evaluate the correlation between PCAT1 levels and various pathological variables. Diagnostic metrics, including the area under the ROC curve, specificity, sensitivity, positive predictive value, negative predictive value, and likelihood ratio, were calculated through ROC curve analysis. Statistical significance was set at p < 0.05. All analyses were repeated in duplicate to ensure reliability.

## 3 Results

### 3.1 Exosomal PCAT1 is significantly upregulated in the serum of early-stage CRC patients, demonstrating high diagnostic value

**Fig. 2:**
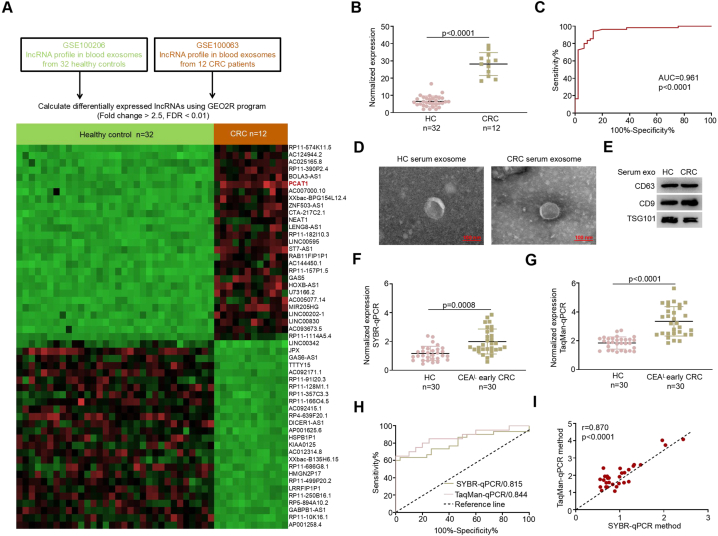
Elevated serum exosomal PCAT1 in early-stage CRC. A Differential expression analysis of lncRNAs was performed using two independent GEO datasets (GSE100206, HC *vs.* GSE100063, CRC). **B** PCAT1 expression levels were significantly higher in CRC patients across both GEO datasets (p < 0.0001). **C** ROC curve analysis of PCAT1 in GEO datasets demonstrated high diagnostic accuracy (AUC = 0.961, p < 0.001). **D** High-resolution transmission electron microscopy images of serum exosomes (scale bar = 100 nm). **E** Western blot analysis detected exosomal markers (CD63, CD9, TSG101) in serum exosomes. **F** and **G** Detection of serum exosomal PCAT1 in CEA-low (CEA^L^) early-stage CRC patients using SYBR-qPCR and TaqMan-qPCR. **H** Diagnostic performance of serum PCAT1 for early CRC detection using two different methods. **I** Comparison and correlation of data from SYBR-qPCR and TaqMan-qPCR methods.

We began our search for a complementary biomarker to CEA by analyzing lncRNA levels in the datasets GSE100063 (CRC, n = 12) and GSE100206 (HC, n = 32). According to our research criteria (Figs. 1), 53 serum exosomal lncRNAs showed differential expression, with 27 upregulated and 26 downregulated (Fig. 2a; Supplemental Table S1). We prioritized upregulated lncRNAs as potential biomarkers, identifying PCAT1 due to its significant change (FC = 3.87; Supplemental Table S1) and high diagnostic performance (AUC = 0.961; Fig. 2b and c).

We then analyzed PCAT1 expression profiles and diagnostic potential using blood samples from 30 early-stage CRC patients with low CEA levels. TEM (Fig. 2d) and Western blot analyses (Fig. 2e) validated the exosomes isolated from serum samples. PCAT1 was significantly upregulated in patients with CEA-low early-stage CRC (Fig. 2f). TaqMan-qPCR confirmed the circulating exosomal PCAT1 expression profile, consistent with SYBR-qPCR findings (p < 0.0001; Fig. 2g). ROC analysis indicated that PCAT1 is an excellent biomarker for early CRC detection (AUC: 0.815 for SYBR-qPCR, 0.844 for TaqMan-qPCR; Fig. 2h). The robustness of our data was further supported by a strong correlation between PCAT1 levels measured by SYBR-qPCR and TaqMan-qPCR (r = 0.870, p < 0.0001; Fig. 2g). These findings suggest that serum exosomal PCAT1 is a promising biomarker for early-stage CRC diagnosis.

### 3.2 Validation of diagnostic potential of circulating exosomal PCAT1 in two independent cohorts

**Fig. 3:**
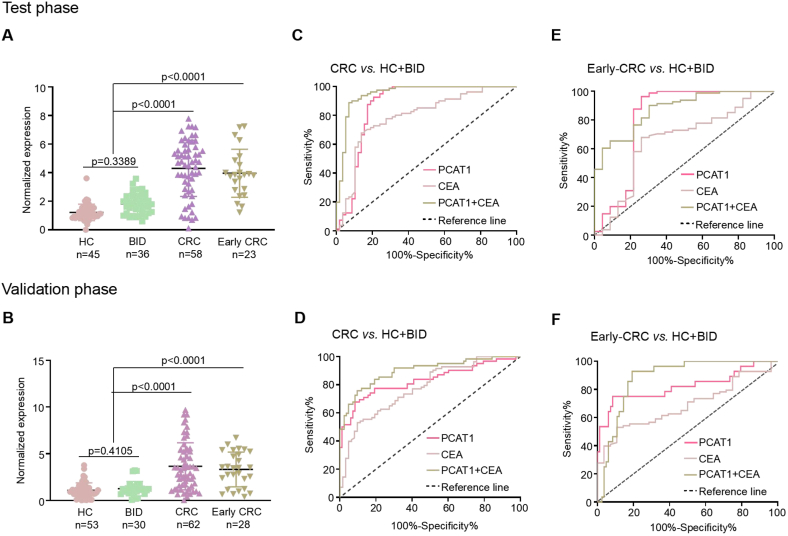
Validation of circulating exosomal PCAT1 in two cohorts. A and **B** Serum exosomal PCAT1 levels were measured in two independent cohorts. **C** and **D** ROC curves showed the diagnostic performance of PCAT1 for CRC patients versus controls in both cohorts. **E** and **F** ROC curves compared PCAT1 for early-stage CRC patients against controls in both cohorts.

The diagnostic utility of PCAT1 for CRC was assessed using sera samples from two independent cohorts. The test cohort included 45 HC, 36 patients with BID, and 58 CRC patients. The validation cohort comprised 53 HC, 30 BID, and 62 CRC patients. In both cohorts, CRC patients showed significantly elevated serum exosomal PCAT1 levels compared to BID and HC (Fig. 3a and b). However, there was no significant difference in PCAT1 expression between the BID and HC groups (Fig. 3a and b). The diagnostic accuracy of PCAT1 and CEA for CRC was then evaluated. Both PCAT1 and CEA showed high diagnostic value for CRC compared to HC and BID (Fig. 3c and d; Table 1). Notably, PCAT1 outperformed CEA in terms of AUC, specificity, and sensitivity in CRC patients (Table 1). Combining both biomarkers significantly enhanced diagnostic performance compared to using each marker individually (Table 1).

**Table 1:** Diagnostic performances of serum exosomal PCAT1, CEA, or both in the test and validation cohort.

	AUC (95%CI)	SEN	SPE	PPV	NPV	LR^+^	LR^-^
CRC *vs.* HC and BID							
PCAT1	0.834 (0.779–0.932)	0.921	0.767	0.857	0.858	3.953	0.103
CEA	0.782 (0.698–0.823)	0.567	0.765	0.767	0.843	2.413	0.566
PCAT1+CEA	0.933 (0.887–0.979)	0.891	0.933	0.962	0.955	13.299	0.117
Early-stage CRC *vs.* HC and BID							
PCAT1	0.836 (0.755–0.911)	0.689	0.933	0.855	0.765	10.284	0.333
CEA	0.666 (0.570–0.762)	0.556	0.687	0.732	0.722	1.776	0.646
PCAT1+CEA	0.881 (0.789–0.921)	0.767	0.823	0.945	0.916	4.333	0.283

Notes: SEN, sensitivity; SPE, specificity; PPV, positive predictive value; NPV, negative predictive value; LR, likelihood ratio.

CEA is not optimal for early-stage CRC diagnosis [10]. Therefore, we evaluated whether PCAT1 had superior diagnostic performance for early-stage CRC compared to CEA. The test cohort included 23 early-stage CRC patients, and the validation cohort included 28 early-stage CRC patients (Dukes stage A/B). PCAT1 levels in serum exosomes from early-stage CRC patients were significantly elevated compared to controls (both p < 0.0001; Fig. 3a and b). Serum exosomal PCAT1 was more effective than CEA in differentiating early-stage CRC patients from controls (Fig. 3e and f; Table 1). PCAT1 also outperformed CEA in terms of likelihood ratios and predictive values (Table 1). These findings confirm the superior diagnostic capacity of PCAT1 for early-stage CRC.

### 3.3 Exosomal PCAT1 serves as a complementary biomarker to CEA

**Fig. 4:**
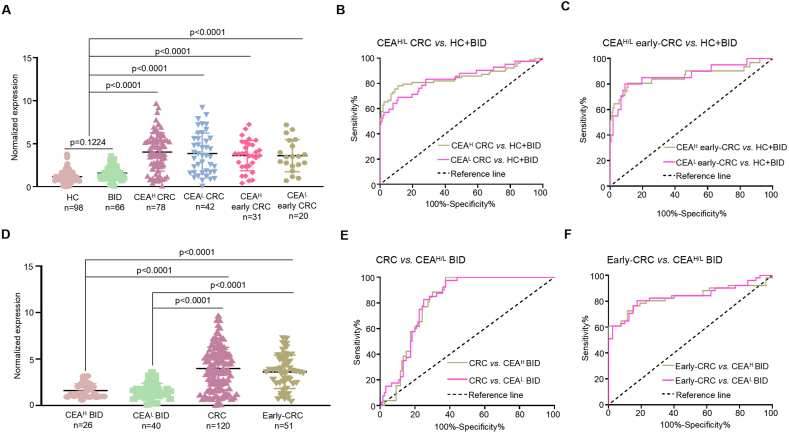
Serum exosomal PCAT1 acts as a complementary biomarker to CEA. A Serum exosomal PCAT1 levels were compared between CEA-high (CEA^H^) and CEA-low (CEA^L^) CRC patients in the test and validation cohorts. **B** ROC curves illustrated the diagnostic value of PCAT1 for CEA^H^ and CEA^L^ CRC patients relative to controls in both cohorts. **C** ROC curves demonstrated PCAT1's diagnostic performance for early-stage CRC patients versus controls. **D** Serum exosomal PCAT1 levels were assessed in CEA^H^ and CEA^L^ BID patients from both cohorts. **E** ROC curves compared PCAT1 levels between CRC patients and CEA^H^ or CEA^L^ BID patients. **F** ROC curves compared early-stage CRC patients with CEA^H^ or CEA^L^ BID patients.

We assessed whether serum exosomal PCAT1 could address CEA's limitations in diagnosing CRC, especially in patients with low CEA levels. Data from two cohorts (test and validation) were merged and categorized into CEA-high and CEA-low groups. As anticipated, serum exosomal PCAT1 levels were markedly elevated in both CEA-high and CEA-low CRC patients compared to controls (p < 0.0001; Fig. 4a). ROC curve analysis confirmed PCAT1 as a promising diagnostic marker for CRC, regardless of CEA status (Fig. 4b; Table 2), indicating that PCAT1 could partly compensate for low CEA levels in CRC diagnosis.

**Table 2:** Diagnostic performances of serum exosomal PCAT1 in CEA-high or CEA-low early CRC detection.

	AUC (95%CI)	SEN	SPE	PPV	NPV	LR^+^	LR^-^
CEA^H^ early-CRC *vs*. HC and BID	0.879 (0.789–0.960)	0.813	0.876	0.565	0.965	6.556	0.213
CEA^L^ early-CRC *vs*. HC and BID	0.888 (0.791–0.965)	0.820	0.923	0.532	0.948	10.649	0.195

Notes: CEA^H^, CEA-high; CEA^L^, CEA-low.

We then evaluated PCAT1's diagnostic potential in early-stage CRC patients. Elevated serum exosomal PCAT1 levels were observed in both CEA-high and CEA-low early-stage CRC patients (p < 0.0001; Fig. 4a). ROC curves showed that PCAT1 provided excellent diagnostic accuracy for early-stage CRC in both CEA-high and CEA-low patients (Fig. 4c; Table 2). Therefore, serum exosomal PCAT1 could be used alone or in combination with other biomarkers to effectively diagnose CEA-low early-stage CRC.

Additionally, low serum exosomal PCAT1 levels were common in most CEA-high BID patients (Fig. 4d). This led us to hypothesize that PCAT1 could differentiate CRC from non-malignant intestinal diseases. ROC analysis supported this, showing that serum exosomal PCAT1 effectively distinguished CRC and early-stage CRC patients from CEA-high and CEA-low BID patients (Fig. 4e and f; Table 2). Thus, serum PCAT1 measurements may enhance the differential diagnosis of CRC in high-risk populations.

### 3.4 Serum exosomal PCAT1 levels correlate with tumor progression and treatment efficacy

We investigated the correlation between serum exosomal PCAT1 levels and clinicopathological features by analyzing the association between PCAT1 expression and clinical characteristics (Table 3). Elevated serum exosomal PCAT1 levels were observed in patients with advanced pathological stages, indicating a possible link between PCAT1 and tumor progression. Higher PCAT1 levels were also associated with larger tumors and more advanced metastasis.

**Table 3:** Pooled analysis of the correlation between serum exosomal PCAT1 levels and clinicopathologic features of CRC patients recruited in the study.

Clinicopathologic features	Total (n = 150)	PCAT1 level		p-value
		Low (n = 75)	High (n = 75)	
Sex				0.1054
Male	97	49	48	
Female	53	26	27	
Age				0.2881
<60	51	26	25	
≥60	99	49	50	
Differentiation				0.3370
Well	60	31	29	
Poor	90	44	46	
Size (cm)				0.0252^a^
<4	71	42	29	
≥4	79	33	46	
Tumor stage (AJCC)				0.0028^b^
Ⅰ-Ⅱ	88	53	35	
Ⅲ-Ⅳ	62	22	40	
Lymphatic metastasis				0.0118^a^
Negative	91	54	37	
Positive	59	21	38	
Vascular metastasis				0.0004^c^
Negative	97	67	30	
Positive	53	8	45	

Note: p-value was analyzed using Chi-square test.

a p < 0.05.

b p < 0.01.

c p < 0.001.

**Fig. 5:**
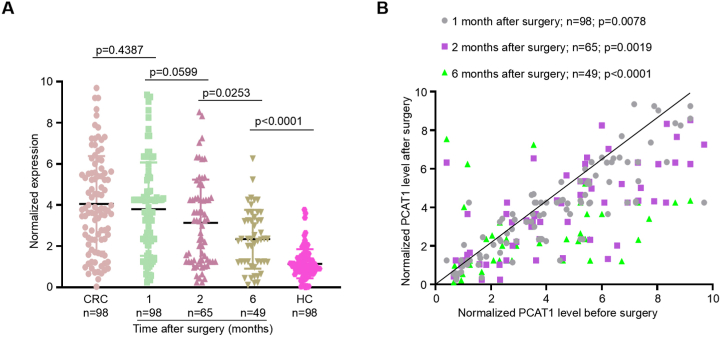
Post-surgical changes in serum exosomal PCAT1 levels. A Comparison of serum exosomal PCAT1 levels before and at 1, 2, and 6 months post-surgery. **B** Scatter plots depicting the variation in serum exosomal PCAT1 levels in matched samples from CRC patients before and after surgery (1, 2, and 6 months).

Identifying reliable biomarkers is crucial for evaluating treatment effectiveness and monitoring disease status during recovery. We assessed serum exosomal PCAT1 levels in pre- and post-operative blood samples from 98 CRC patients who underwent surgical resection, yielding 212 samples. Most patients exhibited a marked decrease in PCAT1 levels within one to six months post-surgery (Fig. 5a and b). These results suggest that serum exosomal PCAT1 may be a useful biomarker for monitoring cancer therapy response and remission. Persistent elevation of serum exosomal PCAT1 post-treatment was linked to a poorer prognosis in CRC patients.

## 4 Discussion

The need for novel serum biomarkers to CRC screening and early diagnosis is critical [16,18,22,25,26]. This study explores the potential of PCAT1 as a circulating exosomal biomarker for CRC, both alone and in combination with CEA. Our findings indicate that serum exosomal PCAT1 offers greater diagnostic accuracy than CEA, especially in early-stage CRC patients with low CEA levels. Additionally, PCAT1 successfully differentiates early-stage CRC patients from those with high CEA levels due to BID, highlighting its promise for early CRC detection.

Currently, there are few reliable biomarkers for early CRC detection [3,6,[27], [28], [29]]. CEA, the most widely used tumor marker for CRC, is elevated in 60%–85 % of cases [8,11], with specificity ranging from 70 % to 90 % but sensitivity limited to 35%–75 % [30,31]. This limited sensitivity emphasizes the need for additional biomarkers. We evaluated the diagnostic potential of the lncRNA PCAT1 in two independent cohorts, particularly for early CRC detection and its complementary role in CEA-low patients. Receiver operating characteristic (ROC) analysis suggests that serum exosomal PCAT1 could function as an independent biomarker for CRC or significantly enhance diagnostic accuracy when combined with CEA. Thus, PCAT1 could serve as a valuable complement to CEA in CRC diagnosis. Previous reports aimed at identification of diagnostic value of PCAT1 in human cancers reported various values ranging from 0.66 to 0.89 in diverse cancers with the best value reported in multiple myeloma [32]. Therefore, we and others consistently suggest that PCAT1 is a reliable biomarker for cancer diagnosis. More importantly, our report provides the first line of evidences that PCAT1 can complementary for CEA in CRC early diagnosis and serves as an auxiliary prognosis monitoring indicator after surgical resection. However, limitations such as a small sample size and a low proportion of CRC patients in our study necessitate further validation in larger multicenter cohorts to confirm the combined diagnostic effectiveness of PCAT1 and CEA, particularly for early-stage CRC.

The prostate cancer associated transcript 1 (PCAT1) was initially demonstrated to exert oncogenic roles in prostate cancer [33]. Further studies revealed its similar roles in a wide array of cancers including endometrial carcinoma [34], colorectal cancer [35], squamous cell carcinoma lung cancer [36], hepatocellular carcinoma [37] and hematological malignancies [38]. PCAT1 was reported to regulate tumor cell proliferation, apoptosis, invasion and metastasis. Among diverse molecular targets of PCAT1, c-Myc and related proliferative pathways is the most potent mechanism of its participation in PCAT1-mediated carcinogenesis [39]. Moreover, matrix metalloproteinases, cyclins, cyclin dependent kinases, EMT-associated proteins are regulated by PCAT1 [[40], [41], [42]]. In addition, PCAT1 regulates expression of multiple miRNAs, which involved in cancer-related pathways including Wnt pathway, Hippo pathway, and NF-κB pathway [[43], [44], [45]]. This suggests that PCAT1 may indirectly regulate these pathways via influencing target miRNAs expression.

Given the high recurrence and metastasis rates in CRC, identifying reliable biomarkers to monitor disease remission is essential [1,46,47]. The significant drop in serum exosomal PCAT1 levels post-surgery suggests that this lncRNA could be a valuable marker for assessing treatment response in CRC patients. Elevated PCAT1 expression is also linked to metastasis and poor prognosis, highlighting its potential as both a diagnostic and prognostic marker. In this study, serum samples were collected at 1, 2, and 6 months post-resection. Our results indicate that serum exosomal PCAT1 provides a rapid assessment of therapeutic outcomes, suggesting its secretion by circulating tumor cells or diffusion from the extracellular environment. Indeed, expression of PCAT1 in clinical tumor samples was extensively elevated compared to that in paracencerous or benign tissues [35,38,48]. Notably, PCAT1 expression was associated with clinical stage, metastasis and survival of patients with diverse types of cancers [34,36,49]. Furthermore, associations between amplification of PCAT1 and overall survival of patients with tumor were consolidated using analysis of TCGA datasets [50,51]. Although serum CEA is commonly used to monitor treatment in advanced disease, its prognostic accuracy is limited [9,10]. Future research should explore the combined use of PCAT1 and CEA to enhance CRC therapy evaluation and predict post-surgical recurrence.

Serum contains a wide range of circulating molecules and cellular materials, such as tumor-derived DNA, ncRNAs, autoantibodies, and metabolites, which can offer crucial insights into tumor progression [[52], [53], [54]]. Understanding the role and significance of potential biomarkers for early CRC diagnosis remains a critical research priority. Our recent validation of PCAT1 as a circulating exosomal marker for CRC underscores the need for further research to develop blood-based biomarker methods that complement existing CRC screening techniques.

## 5 Conclusion

Our findings indicate that serum exosomal PCAT1 has strong potential as an independent biomarker for early CRC detection and may also enhance the diagnostic utility of the established biomarker CEA.

## CRediT authorship contribution statement

**Jinghe Cao:** Investigation, Data curation. **Wei Chao:** Investigation, Data curation. **Jiansheng Zhang:** Visualization, Software, Resources. **Jiajia Mao:** Supervision, Resources, Investigation. **Jianchao Zeng:** Supervision, Resources, Formal analysis, Data curation. **Delan Luo:** Validation, Software, Methodology, Data curation. **Shishun Huang:** Visualization, Validation, Supervision, Formal analysis. **Jiashu Li:** Writing – original draft, Software, Formal analysis. **Baoyu He:** Writing – review & editing, Writing – original draft, Investigation, Formal analysis. **Hongli Pan:** Writing – original draft, Visualization, Conceptualization.

## Data availability statement

The original contributions presented in the study are included in the article/Supplementary Material, and further inquiries can be directed to the corresponding author.

## Ethics statement

Approval for the study was obtained from the Research Ethics Committee of the Fourth Affiliated Hospital of Guangxi Medical University and the Affiliated Hospital of Jining Medical University (approval number: PJ20180155). Informed consent was obtained from participants, according to the committee’ regulations.

## Funding

This work was supported by grant from the 10.13039/501100001809National Natural Science Foundation of China (81802910, 82273069), Project funded by 10.13039/501100002858China Postdoctoral Science Foundation (2022M711322), Shandong Postdoctoral innovation project (SDCX-ZG-202201002), Self-funded scientific research project of the Guangxi 10.13039/100018696Health Commission (Z20190296), the 10.13039/501100004607Natural Science Foundation of Guangxi Province (2018GXNSFAA138029), Ph.D. Research Foundation of the Affiliated Hospital of 10.13039/501100008853Jining Medical University (2022-BS-003), Research Fund for Lin He's Academician Workstation of New Medicine and Clinical Translation in 10.13039/501100008853Jining Medical University (JYHL2022FZD04).

## Declaration of competing interest

The authors declare that they have no known competing financial interests or personal relationships that could have appeared to influence the work reported in this paper.

## References

[1] Biller L.H., Schrag D. (2021). Diagnosis and treatment of metastatic colorectal cancer: a review. JAMA.

[2] Sinicrope F.A. (2022). Increasing incidence of early-onset colorectal cancer. N. Engl. J. Med..

[3] Davidson K.W. (2021). Screening for colorectal cancer: US preventive services task force recommendation statement. JAMA.

[4] Wu Z. (2020). Colorectal cancer screening methods and molecular markers for early detection. Technol. Cancer Res. Treat..

[5] Jin M., Frankel W.L. (2018). Lymph node metastasis in colorectal cancer. Surg Oncol Clin N Am.

[6] Chan S.C.H., Liang J.Q. (2022). Advances in tests for colorectal cancer screening and diagnosis. Expert Rev. Mol. Diagn.

[7] Maida M. (2017). Screening of colorectal cancer: present and future. Expert Rev. Anticancer Ther..

[8] Goldstein M.J., Mitchell E.P. (2005). Carcinoembryonic antigen in the staging and follow-up of patients with colorectal cancer. Cancer Invest..

[9] Kawamura H. (2022). Prognostic role of carcinoembryonic antigen and carbohydrate antigen 19-9 in stage IV colorectal cancer. Anticancer Res..

[10] Kim S.S., Donahue T.R., Girgis M.D. (2018). Carcinoembryonic antigen for diagnosis of colorectal cancer recurrence. JAMA.

[11] Li L. (2021). Fecal CEA has an advantage in the diagnosis of colorectal cancer at early stage. Cancer Control.

[12] He B. (2022). Long noncoding RNA LINC00930 promotes PFKFB3-mediated tumor glycolysis and cell proliferation in nasopharyngeal carcinoma. J. Exp. Clin. Cancer Res..

[13] He B. (2016). Epstein-Barr virus-encoded miR-BART6-3p inhibits cancer cell metastasis and invasion by targeting long non-coding RNA LOC553103. Cell Death Dis..

[14] Ou C. (2020). Targeting YAP1/linc00152/FSCN1 signaling Axis prevents the progression of colorectal cancer. Adv. Sci..

[15] Liao Z. (2021). The emerging landscape of long non-coding RNAs in colorectal cancer metastasis. Front. Oncol..

[16] He B. (2017). Serum long non-coding RNAs MALAT1, AFAP1-AS1 and AL359062 as diagnostic and prognostic biomarkers for nasopharyngeal carcinoma. Oncotarget.

[17] Pan H. (2020). Serum long non-coding RNA LOC553103 as non-specific diagnostic and prognostic biomarker for common types of human cancer. Clin. Chim. Acta.

[18] He X., Ou C. (2017). Exosome-derived microRNAs in cancer progression: angel or devil?. J. Thorac. Dis..

[19] Li X. (2022). The role of cancer stem cell-derived exosomes in cancer progression. Stem Cells Int.

[20] He X. (2021). Emerging roles of exosomal miRNAs in diabetes mellitus. Clin. Transl. Med..

[21] Peng L. (2021). Emerging role of cancer-associated fibroblasts-derived exosomes in tumorigenesis. Front. Immunol..

[22] Ding X.Z. (2021). Serum exosomal lncRNA DLX6-AS1 is a promising biomarker for prognosis prediction of cervical cancer. Technol. Cancer Res. Treat..

[23] Lan F. (2021). Serum exosomal lncRNA XIST is a potential non-invasive biomarker to diagnose recurrence of triple-negative breast cancer. J. Cell Mol. Med..

[24] Zhao S. (2020). Tumor-derived exosomal miR-934 induces macrophage M2 polarization to promote liver metastasis of colorectal cancer. J. Hematol. Oncol..

[25] Wang Y. (2020). Application of artificial intelligence to the diagnosis and therapy of colorectal cancer. Am. J. Cancer Res..

[26] Nie H. (2021). Exosomal long non-coding RNAs: emerging players in cancer metastasis and potential diagnostic biomarkers for personalized oncology. Genes Dis.

[27] Burnett-Hartman A.N. (2021). An update on the epidemiology, molecular characterization, diagnosis, and screening strategies for early-onset colorectal cancer. Gastroenterology.

[28] Zygulska A.L., Pierzchalski P. (2022). Novel diagnostic biomarkers in colorectal cancer. Int. J. Mol. Sci..

[29] He B. (2021). Hsa_circ_001659 serves as a novel diagnostic and prognostic biomarker for colorectal cancer. Biochem. Biophys. Res. Commun..

[30] Engarås B. (2001). CEA, CA 50 and CA 242 in patients surviving colorectal cancer without recurrent disease. Eur. J. Surg. Oncol..

[31] Suresh Babu M.C. (2017). Colorectal cancer presenting as bone metastasis. J Cancer Res Ther.

[32] Ghafouri-Fard S., Dashti S., Taheri M. (2020). PCAT1: an oncogenic lncRNA in diverse cancers and a putative therapeutic target. Exp. Mol. Pathol..

[33] Walsh A.L. (2014). Long noncoding RNAs and prostate carcinogenesis: the missing 'linc'?. Trends Mol. Med..

[34] Zhao X. (2019). PCAT1 is a poor prognostic factor in endometrial carcinoma and associated with cancer cell proliferation, migration and invasion. Bosn. J. Basic Med. Sci..

[35] Ge X. (2013). Overexpression of long noncoding RNA PCAT-1 is a novel biomarker of poor prognosis in patients with colorectal cancer. Med. Oncol..

[36] Shi W.H. (2015). Upregulation of the long noncoding RNA PCAT-1 correlates with advanced clinical stage and poor prognosis in esophageal squamous carcinoma. Tumour Biol.

[37] Yan T.H. (2015). Prognostic significance of long non-coding RNA PCAT-1 expression in human hepatocellular carcinoma. Int. J. Clin. Exp. Pathol..

[38] Shen X. (2017). Upregulated lncRNA-PCAT1 is closely related to clinical diagnosis of multiple myeloma as a predictive biomarker in serum. Cancer Biomark.

[39] Prensner J.R. (2014). The long non-coding RNA PCAT-1 promotes prostate cancer cell proliferation through cMyc. Neoplasia.

[40] Wang A.H. (2019). LncRNA PCAT-1 regulated cell proliferation, invasion, migration and apoptosis in colorectal cancer through targeting miR-149-5p. Eur. Rev. Med. Pharmacol. Sci..

[41] Ding C. (2019). LncRNA PCAT-1 plays an oncogenic role in epithelial ovarian cancer by modulating cyclinD1/CDK4 expression. Int. J. Clin. Exp. Pathol..

[42] Zhang S. (2022). lncRNA PCAT1 might coordinate ZNF217 to promote CRC adhesion and invasion through regulating MTA2/MTA3/Snai1/E-cadherin signaling. Cell Mol Biol (Noisy-le-grand).

[43] Zhang F. (2017). Long noncoding RNA PCAT1 regulates extrahepatic cholangiocarcinoma progression via the Wnt/β-catenin-signaling pathway. Biomed. Pharmacother..

[44] Wang R. (2020). Long non-coding RNA PCAT1 drives clear cell renal cell carcinoma by upregulating YAP via sponging miR-656 and miR-539. Cell Cycle.

[45] Shen X. (2020). PCAT-1 promotes cell growth by sponging miR-129 via MAP3K7/NF-κB pathway in multiple myeloma. J. Cell Mol. Med..

[46] Hao Y. (2022). Nuclear translocation of p85β promotes tumorigenesis of PIK3CA helical domain mutant cancer. Nat. Commun..

[47] Yang H. (2020). TP53 mutation influences the efficacy of treatment of colorectal cancer cell lines with a combination of sirtuin inhibitors and chemotherapeutic agents. Exp. Ther. Med..

[48] Zhang X. (2018). The lncRNA PCAT1 is correlated with poor prognosis and promotes cell proliferation, invasion, migration and EMT in osteosarcoma. OncoTargets Ther..

[49] Cui W.C., Wu Y.F., Qu H.M. (2017). Up-regulation of long non-coding RNA PCAT-1 correlates with tumor progression and poor prognosis in gastric cancer. Eur. Rev. Med. Pharmacol. Sci..

[50] Shang Z. (2019). LncRNA PCAT1 activates AKT and NF-κB signaling in castration-resistant prostate cancer by regulating the PHLPP/FKBP51/IKKα complex. Nucleic Acids Res..

[51] Sur S. (2019). Depletion of PCAT-1 in head and neck cancer cells inhibits tumor growth and induces apoptosis by modulating c-Myc-AKT1-p38 MAPK signalling pathways. BMC Cancer.

[52] Li S. (2016). Serum microRNA-21 as a potential diagnostic biomarker for breast cancer: a systematic review and meta-analysis. Clin. Exp. Med..

[53] Maurya P. (2007). Proteomic approaches for serum biomarker discovery in cancer. Anticancer Res..

[54] Liu L. (2020). Serum SYPL1 is a promising diagnostic biomarker for colorectal cancer. Clin. Chim. Acta.

