# Supplementary figures and images for: Circulating exosomal PCAT1 as a complement of carcinoembryonic antigen for early colorectal cancer diagnosis
Source: Heliyon. 2024 Oct 11;10(20):e39264. doi: 10.1016/j.heliyon.2024.e39264 (PMC11620264; doi:10.1016/j.heliyon.2024.e39264)

Uncropped WB images of Fig. 2E

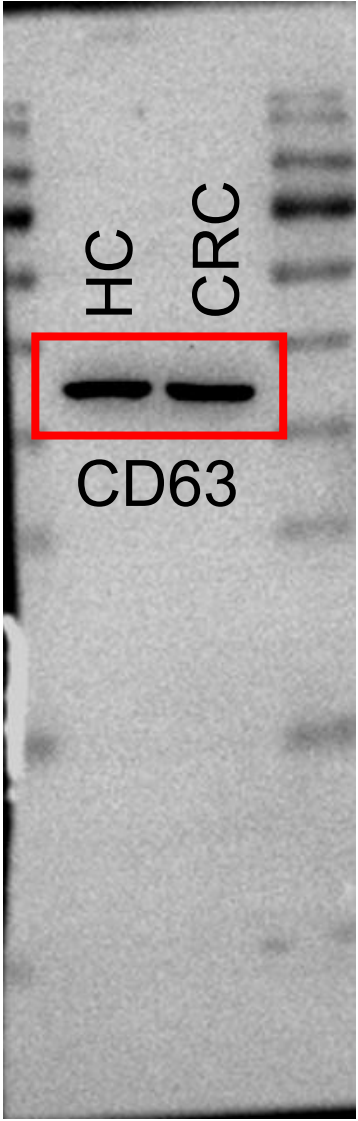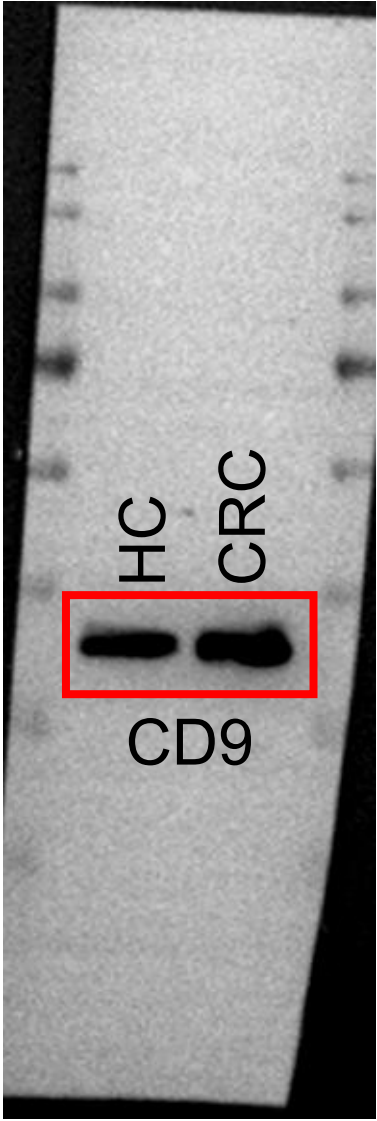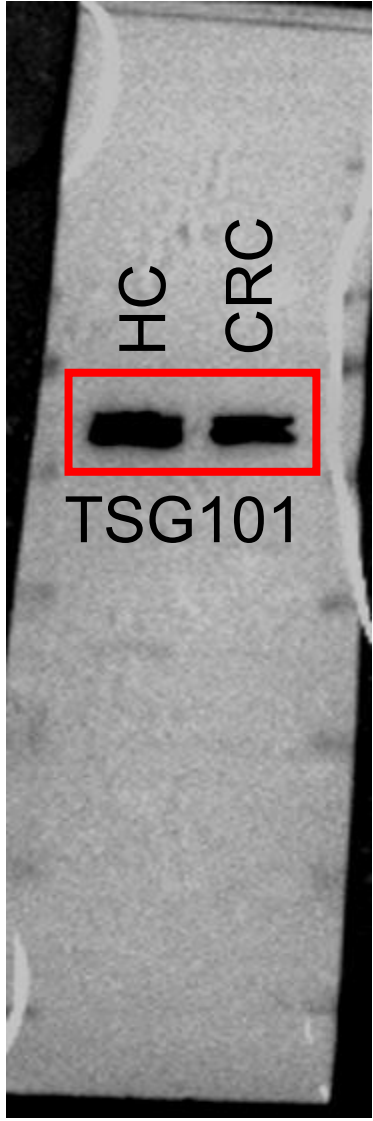

Supplement: Multimedia component 3 [file mmc3.pdf]
